# Management and Long-Term Outcomes of Post-traumatic Chronic Osteomyelitis in Long Bones: Cierny-Mader Types III and IV

**DOI:** 10.7759/cureus.77735

**Published:** 2025-01-20

**Authors:** Fırat Ozan, Kürşat Tuğrul Okur, Koray Özdemir, Mehmet Çavuş, Hatice Karaman, Ilhami Celik

**Affiliations:** 1 Orthopaedics and Traumatology, The University of Health Sciences, Kayseri City Training and Research Hospital, Kayseri, TUR; 2 Hand Surgery, The University of Health Sciences, Kayseri City Training and Research Hospital, Kayseri, TUR; 3 Pathology, The University of Health Sciences, Kayseri City Training and Research Hospital, Kayseri, TUR; 4 Infectious Diseases and Clinical Microbiology, The University of Health Sciences, Kayseri City Training and Research Hospital, Kayseri, TUR

**Keywords:** chronic osteomyelitis, infected bone defects, long bone, post-traumatic osteomyelitis, treatment

## Abstract

Background

This study describes the long-term clinical and functional outcomes of patients with post-traumatic Cierny-Mader (C-M) Type III and IV chronic osteomyelitis (CO), managed by considering individual patient differences.

Methods

Twenty patients who developed CO of the long bones after trauma were included in this study. Data on the demographic characteristics of the patients, clinical and radiological characteristics, and surgical methods applied were collected. The classification system defined by C-M was used for CO classification. The Short Form-36 (SF-36) quality of life scale was used to evaluate the functional outcomes and quality of life of the patients at the end of follow-up.

Results

The participants included 16 males and four females, with an average age of 39.3 ± 14.5 years. The mean duration of CO was 6.8 ± 7.5 years. The anatomical location of the CO was in the tibia in 15 patients, in the femur in four, and in the radius in one. The mean follow-up time after CO reconstruction was 4.5 ± 1.05 years. According to the C-M anatomical classification, there were nine patients with Type III and 11 with Type IV. According to the C-M physiological classification, there was one patient with Class A, 16 with Class B1, and three with Class B2. Different combinations of surgical procedures were performed on each patient. The average number of surgical interventions performed on the patients was 3.1 ± 1.1. Culture growth was detected in 13 patients. At the end of follow-up, the patients’ SF-36 scores were lower than those in the normal population.

Conclusion

Due to the varied histories of CO and individual differences, it is quite challenging to plan a standard treatment procedure for CO in clinical practice. Successful treatment can be achieved with a long-term multidisciplinary approach and individualized, well-planned treatment methods.

## Introduction

Chronic osteomyelitis (CO) is a progressive disease characterized by infectious and inflammatory processes that damage bone tissue, caused by microorganisms [1,2]. Osteomyelitis is categorized as acute, subacute, or chronic [1,3]. In terms of pathogenicity, CO occurs a few months after a bone infection, characterized by bone destruction and sequestration [1-4]. Currently, osteomyelitis often develops due to post-traumatic causes and is implant-related, affecting patients with comorbidities [4-6]. Implants or open fractures favor the entry of microorganisms [1,2,5-7]. Although all bones may be involved, osteomyelitis is mainly seen in the lower extremities, with the tibia being the most common site where CO develops after trauma [2,5-7].

Despite the development of drug therapies, surgical options, and supportive treatments, CO treatment remains a challenge [2,3,8-10]. CO tends to develop complications such as pain, disability, uncontrolled infection, delayed recovery, nonunion, repeated surgical procedures, and prolonged hospital stays, which can significantly reduce an individual’s quality of life [2,7-9,11]. In most cases, such infected limbs are considered unrecoverable [1,2,12,13]. The infection is either left untreated, or amputation is offered as a treatment option [1,2,12,13].

Many treatment procedures, including some unique treatments, have been described for CO [1,3,4,7-16]. Treatment requires the isolation of pathogens if possible, appropriate antibiotic therapy, careful debridement of all infected and necrotic tissue, and subsequent reconstruction of bone and soft tissues [1,2,6-10,15,16]. Inadequate debridement may cause recurrence and chronicity of the disease, while wider resection will cause defects in bone and soft tissue that require reconstruction [1,7,10,12-17]. Therefore, maintaining balance in the treatment process is very important [10,13,16]. In this study, we describe the long-term clinical and functional outcomes of patients with post-traumatic Cierny-Mader (C-M) Type III and IV CO, who were managed considering the individual differences of the patients, leading to successful outcomes.

## Materials and methods

Patients

Data from 20 patients who developed CO in the long bones after trauma and were treated in at least two stages were analyzed retrospectively. CO was defined as symptom duration of at least six months accompanied by radiological, microbiological, and hematological findings, as well as clinical features such as sinus tracts in the extremity, abscess, pus, pain, swelling, and redness. Exclusion criteria included patients with non-posttraumatic osteomyelitis, acute osteomyelitis, diabetic foot infections, presence of tumoral disease, and those with a history of implant allergies.

Data collection and evaluation

Data on the demographic characteristics of the patients, clinical, radiological, and laboratory characteristics, CO type, anatomical location of the involvement area, previous treatments, soft tissue condition, type of stabilization applied, surgical methods applied, microbiological culture results, antibiotic treatment, duration of treatment, length of hospital stay, and complications developed were collected.

All patients were preoperatively evaluated by plain radiographs, CT, and contrast-enhanced MRI to determine the likely extent of bone and soft tissue debridement and the methods of fixation to be used. In some patients, leukocyte-labeled Technetium-99m imaging (LLTI) was used. Laboratory assessments included a complete blood count, procalcitonin (PCT), erythrocyte sedimentation rate (ESR), C-reactive protein (CRP), hepatic and renal function tests, blood glucose, electrolytes, and a bleeding profile. Intraoperative microbiological deep tissue cultures were taken, and antibiotherapy was determined based on culture antibiogram results.

The physiology- and anatomy-based osteomyelitis classification system described by C-M was used to assist in planning the surgical strategy. According to this classification system, CO is anatomically divided into four types: medullary (type I), superficial (type II), localized (type III), and diffuse (type IV). Patients are also divided into groups according to their physiological status: Class A (no comorbidities), Class B1 (local compromise in the affected limb), Class B2 (systemic compromise), and Class C (too weak to be operated). All surgical procedures and follow-up assessments were performed by the same surgeon.

The Short Form-36 (SF-36) quality of life scale was used to evaluate the functional outcomes and quality of life of the patients at the end of follow-up. SF-36 provides the opportunity to evaluate the health status of an individual using eight sub-parameters, including physical function (PF), physical role (PR), bodily pain (BP), general health (GH), vitality (VT), social functioning (SF), emotional role (ER), and mental health (MH) [18].

Ethical approval

The University of Health Sciences Kayseri City Training and Research Hospital Clinical Research Ethics Committee approved the study protocol (approval No.29.07.2021/453), informed consent was obtained from each patient, and the study was conducted in accordance with the principles of the Declaration of Helsinki.

Statistical analysis

All data analyses were performed using IBM Statistical Package for Social Sciences v. 22.0 (IBM Corp., Armonk, USA). Percentages and means with standard deviations (SDs) were determined for categorical data and continuous variables, respectively. The normality of the data was tested using the Shapiro-Wilk test. Preoperative and postoperative laboratory results were compared using the Wilcoxon signed-rank test, and a P-value < 0.05 was considered statistically significant.

## Results

There were 16 males and four females with an average age of 39.3 ± 14.5 (range, 16-60) years. The mean duration of CO was 6.8 ± 7.5 (range, 1-25) years. CO was anatomically located in the tibia in 15 (75%) patients, in the femur in four (20%) patients, and in the radius in one (5%) patient. The mean length of hospitalization was 6.1 ± 4.01 (range, 2-16) weeks. The mean follow-up time after CO reconstruction was 4.5 ± 1.05 (range, 2-6) years (Table 1).

**Table 1:** Patients' demographic characteristics. n = number; CO = chronic osteomyelitis.

Patient characteristics		
Number of patients		20
Age, years, mean ± SD (range)		39.3 ± 14.5 (16-60)
Sex, n (%)	Female	4 (20)
	Male	16 (80)
Side of involvement, n (%)	Right	8 (40)
	Left	12 (60)
Duration of CO, years, mean ± SD (range)		6.8 ± 7.5 (1-25)
Length of hospitalization, weeks, mean ± SD (range)		6.1 ± 4.01 (2-16)
Number of surgical procedures, mean ± SD (range)		3.1 ± 1.1 (2-6)
Follow-up, years, mean ± SD (range)		4.5 ± 1.05 (2-6)

**Figure 1:**
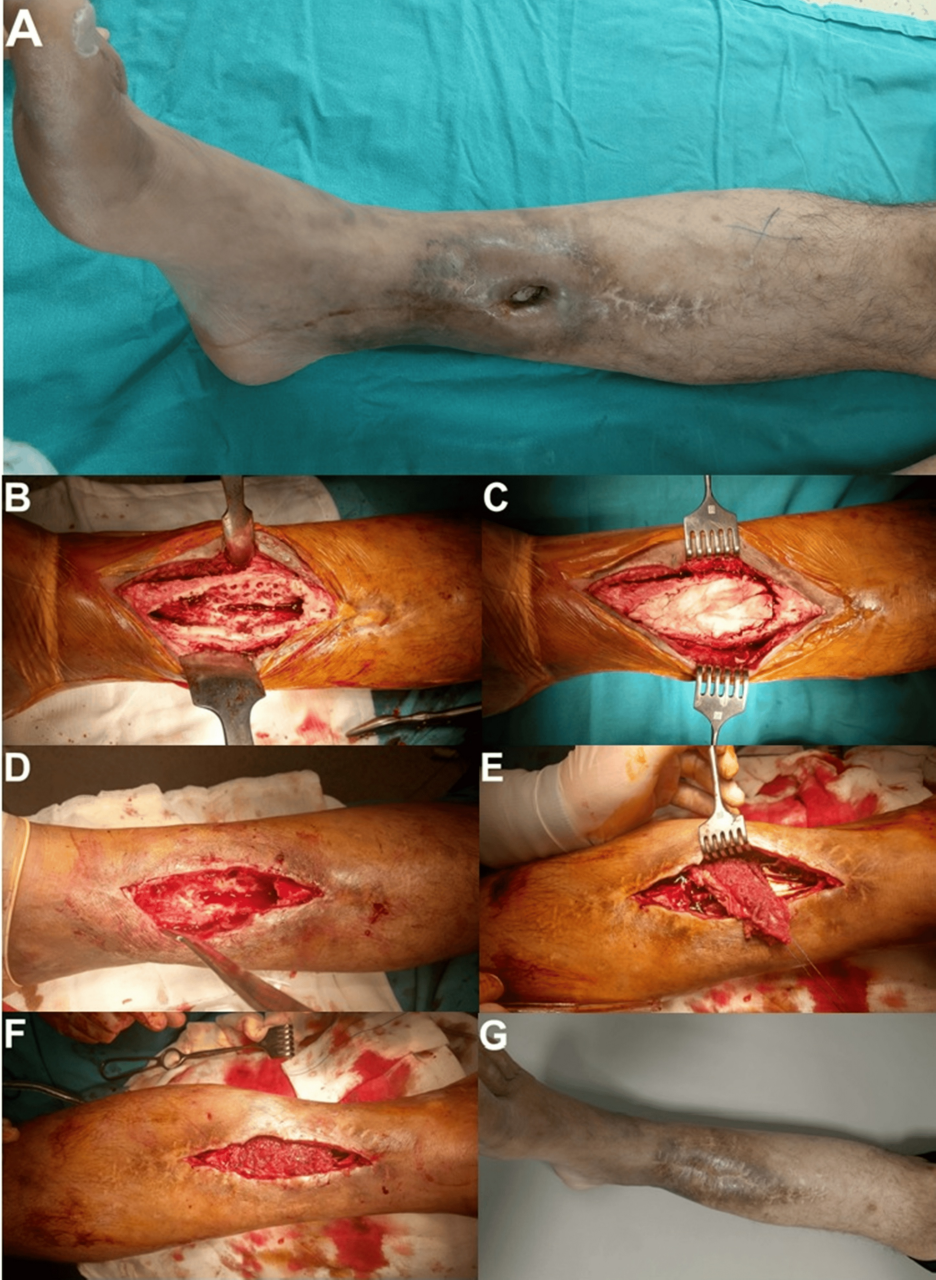
A 52-year-old male patient with a history of Cierny-Mader Type III B1 chronic osteomyelitis in the right tibia for 10 years. (A) The appearance of chronic osteomyelitis. (B, C) Intraoperative images of the implanted antibiotic-impregnated bone cement spacer after fenestration, debridement, and drilling of the osteomyelitic area. (D, E, F) Images of the patient after removal of the antibiotic-impregnated bone cement spacer, bone autografting, and soleus muscle transfer for the skin defect in the patient's subsequent follow-up. (G) The appearance of the patient’s healed extremity after six years of follow-up. C-M: Cierny and Mader; B1: local compromise in the affected limb. Image Credits: Corresponding Author.

**Figure 2:**
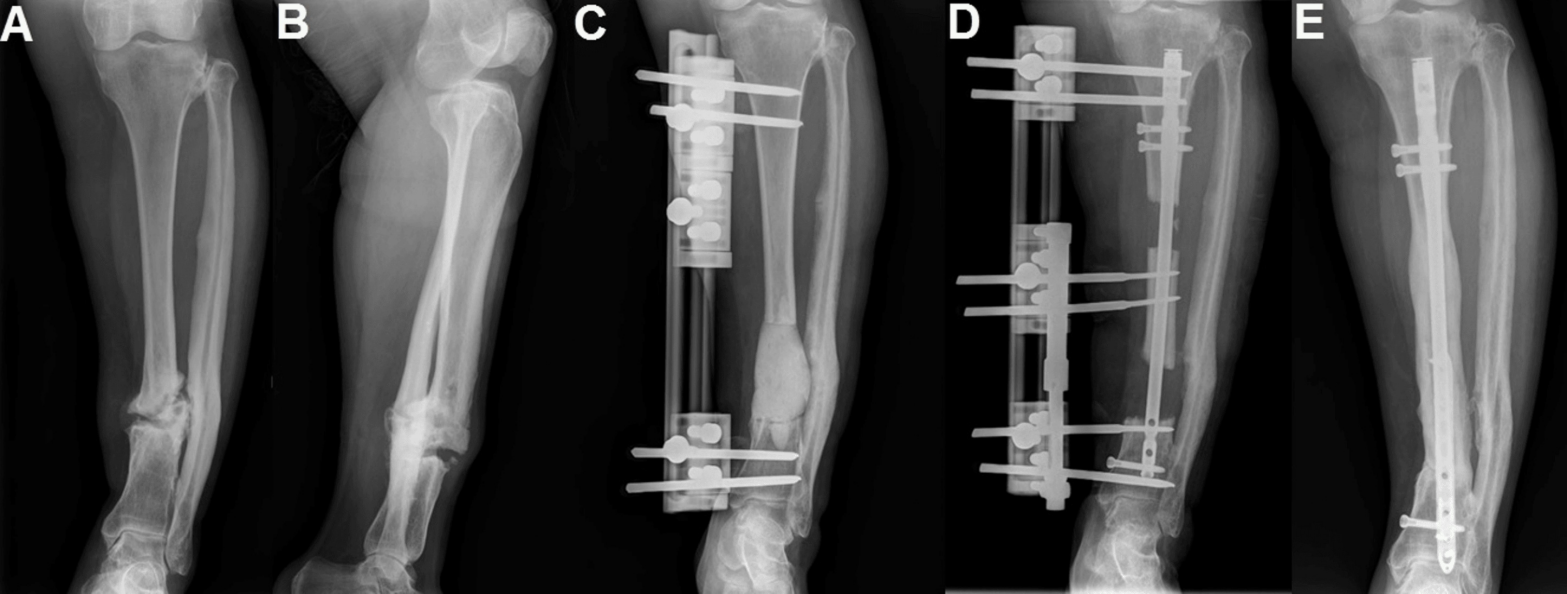
A 44-year-old female patient with a 12-year history of left tibia Cierny-Mader type IV B1 chronic osteomyelitis. (A, B) Anteroposterior and lateral radiographs of the patient. (C) Image of the implanted antibiotic-impregnated bone cement spacer after segmental block resection of the tibia. (D) Radiographic image of the patient after removal of the bone cement and performance of bifocal bone transport in subsequent follow-ups. (E) Radiographic image of the patient, healed, after four years of follow-up. C-M: Cierny and Mader; B1: local compromise in the affected limb. Image Credits: Corresponding Author.

**Figure 3:**
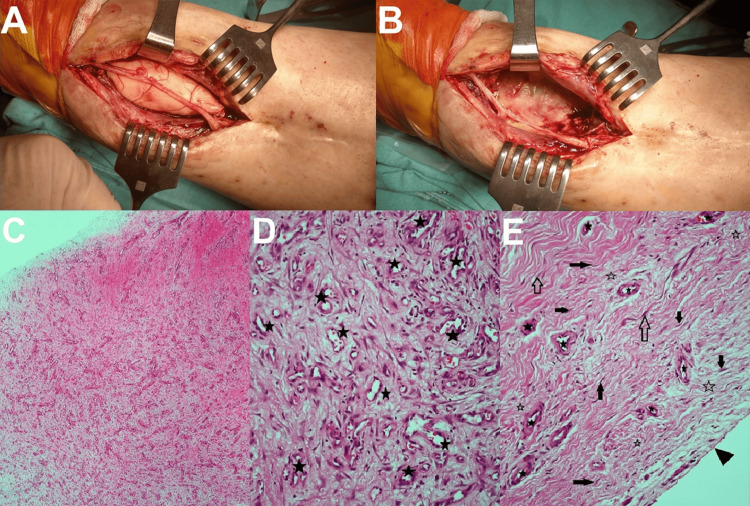
Induced membrane technique and histopathological images. (A) Intraoperative image of a patient with chronic osteomyelitis of the left tibia, undergoing the Masquelet technique with placement of an antibiotic-impregnated bone cement spacer after bone resection. (B) At the end of the follow-up, the intraoperative image shows the clean area with the membrane formed around the spacer after the removal of the bone cement. (C) Histopathological image showing cellular dominance in the stroma of the membrane formed around the bone cement spacer (haematoxylin and eosin staining; magnification, ×40). (D, E) Histopathological images showing a richly vascularized structure (asterisk) covered with a synovial membrane (arrowhead) consisting of fibroblasts (arrow), myofibroblasts (hollow arrow), and collagen fibers (hollow asterisk) (haematoxylin and eosin staining; magnification, ×200). Image Credits: Corresponding Author

According to the C-M anatomic classification, there were nine (45%) patients with type III and 11 (55%) with type IV. According to the C-M physiologic classification, there was one (5%) patient of Class A, 16 (80%) of Class B1, and three (15%) of Class B2. Regarding etiology, CO developed after trauma in all patients, of which seven (35%) had CO secondary to closed fractures, and the other 13 (65%) had CO secondary to open fractures. All the patients underwent surgery due to their fractures. All patients had at least one sinus tract with discharge in their extremities, and nine (45%) patients had soft tissue defects. Six (30%) patients had peroneal nerve deficits. Seven (35%) patients had contractures in the bone-related joints with CO. The patients underwent an average of 3.1 ± 1.1 (range, 2-6) surgical interventions, including surgical debridement, during the treatment process. Different combinations of surgical procedures were performed on each patient (Figures 1-3).

The surgical procedures performed included antibiotic-impregnated polymethylmethacrylate (PMMA) bone cement spacer (AIBCS) implantation, induced membrane technique application (Masquelet technique), monolateral-Ilizarov external fixation application, autograft-allograft application, soleus muscle flap, local flap application, free fascia transfer, intramedullary nailing application, free nonvascularized and vascularized fibular grafting, plate osteosynthesis, monofocal-bifocal bone transport, and bone lengthening (Table 2). 

**Table 2:** Patient characteristics and clinical outcomes. C–M: Cierny and Mader; A: no comorbidities; B1: local compromise in the affected limb; B2: systemic compromise; AIBCS: antibiotic-impregnated bone cement spacer; PMMA: polymethylmethacrylate; MEF: monolateral external fixation; IMT: induced membrane technique; AUG: autograft; SMF: soleus muscle flap; PO: plate osteosynthesis; IEF: Ilizarov external fixation; BBT: bifocal bone transport; IMN: intramedullary nailing; ALG: allograft; MBT: monofocal bone transport; FVFG: free vascularized fibular grafting; FNVFG: free non-vascularized fibular grafting; LF: local flap; FFT: free fascia transfer; BL: bone lengthening.

Patients	Age	Sex	Site	Type of fracture	C–M classification	Duration of chronic osteomyelitis (years)	Length of hospitalization (weeks)	Follow-up (years)	Number of surgery	Applied surgical procedures	Outcomes
1	52	Male	Tibia	Closed	III Bl	10	5	6	4	AIBCS, MEF, IMT, AUG, SMF	Cured
2	50	Male	Tibia	Open	IV Bl	2	3	4	2	AIBCS, MEF, IMT, PO	Cured
3	43	Male	Tibia	Open	IV Bl	1	12	4	4	AIBCS, IEF, IMT, AUG, PO	Cured
4	44	Male	Tibia	Open	III Bl	3	3	4	2	AIBCS, MEF	Cured
5	60	Female	Tibia	Closed	III Bl	2	2	4	2	AIBCS, MEF	Cured
6	55	Male	Femur	Closed	IV Bs	25	4	3	4	AIBCS, MEF	Cured
7	44	Female	Tibia	Open	IV Bl	12	16	4	4	AIBCS, MEF, IMT, BBT, IMN, PO	Cured
8	44	Male	Tibia	Closed	III Bl	20	6	3	4	AIBCS, IMT, MEF, AUG, ALG, SMF, IMN	Cured
9	50	Male	Femur	Closed	IV Bl	20	4	3	2	AIBCS, MEF, IMT, AUG, PO	Cured
10	25	Male	Tibia	Open	IV Bl	5	6	4	6	AIBCS, IEF, IMT, MBT, PO	Cured
11	16	Male	Radius	Open	III A	1	3	3	2	AIBCS, MEF, IMT, FVFG, PO	Cured
12	43	Male	Femur	Closed	III Bs	5	4	5	3	AIBCS, MEF	Cured
13	16	Male	Tibia	Open	IV Bl	3	2	3	2	AIBCS, MEF	Cured
14	59	Female	Tibia	Open	IV Bs	15	8	3	4	AIBCS, MEF, IMT, FNVFG, PO, LF	Cured
15	31	Male	Femur	Open	III Bl	4	6	3	3	AIBCS, MEF	Cured
16	25	Male	Tibia	Closed	IV Bl	1	12	3	3	AIBCS, MEF, IMT, AUG, LF	Cured
17	18	Male	Tibia	Open	IV Bl	1	8	2	2	AIBCS, MEF, IMT, FFT, IMN	Cured
18	45	Male	Tibia	Open	III Bl	4	3	2	2	AIBCS, MEF	Cured
19	47	Male	Tibia	Open	III Bl	1	3	2	3	AIBCS, IMT, MEF, AUG, ALG, IMN	Cured
20	20	Female	Tibia	Open	IV Bl	2	12	5	4	AIBCS, IEF, MBT, BL, IMN	Cured

Regarding preoperative and postoperative blood parameters, the average WBC count was 9.09 ± 2.6 × 10^3^/µL preoperatively and 7.4 ± 1.7 × 10^3^/µL postoperatively (p = 0.049), whereas the mean sedimentation level was 26.3 ± 24.7 mm/h and 13.1 ± 15.4 mm/h, respectively (p = 0.066). Mean CRP level was 39.8 ± 56.6 mg/L and 11.2 ± 12.6 mg/L, respectively (p = 0.015), and mean serum PCT level was 0.28 ± 0.8 µg/L and 0.04 ± 0.02 µg/L, respectively (p = 0.117) (Table 3).

**Table 3:** Comparison of preoperative and postoperative laboratory results. ESR: Erythrocyte sedimentation rate; CRP: C-reactive protein; PCT: Procalcitonin. *Wilcoxon signed ranks test used for statistical analysis.

Variables	Preoperative	Postoperative	P-value*
WBC (10^3^/µL), mean ± SD	9.09 ± 2.6	7.4 ± 1.7	0.049
ESR (mm/hr), mean ± SD	26.3 ± 24.7	13.1 ± 15.4	0.066
CRP (mg/L), mean ± SD	39.8 ± 56.6	11.2 ± 12.6	0.015
PCT (µg/L), mean ± SD	0.28 ± 0.8	0.04 ± 0.02	0.117

Culture growth was detected in 13 (65%) patients. Polymicrobial growth was observed in seven (53.8%) cultures. Staphylococcus aureus was the most common infectious agent. The microbial culture results of the patients and the antibiotherapy they received during hospitalization and after discharge are summarized in Table 4.

**Table 4:** Microbial culture results and antibiotic treatments of the patients. MSSA: Methicillin-sensitive Staphylococcus aureus; MRSA: Methicillin-resistant Staphylococcus aureus.

Patients	Organisms	Parenteral treatment	Oral treatment
1	Escherichia coli, Pseudomonas aeruginosa, Enterococcus faecalis	Ertapenem 1gr (1 times/day), Teicoplanin 400 mg (1 times/day), Ampicillin/sulbactam 1gr (4 times/day)	Ciprofloxacin 750 mg (2 times/day), Sodium Fucidate 500 mg (3 times/day)
2	Negative	Teicoplanin 400 mg (1 times/day)	Ciprofloxacin 750 mg (2 times/day), Sodium Fucidate 500 mg (3 times/day)
3	Pseudomonas oleovorans, Enterococcus faecium	Ertapenem 1gr (1 times/day), Teicoplanin 400 mg (1 times/day)	Ciprofloxacin 750 mg (2 times/day), Sodium Fucidate 500 mg (3 times/day)
4	Staphylococcus aureus (MSSA +)	Ampicillin/sulbactam 1gr (4 times/day)	Trimethoprim/sulfamethoxazole 800/160 mg (2 times/day)
5	Staphylococcus aureus (MRSA +)	Teicoplanin 400 mg (1 times/day)	Ciprofloxacin 750 mg (2 times/day), Sodium Fucidate 500 mg (3 times/day)
6	Klebsiella pneumoniae	Meropenem 1 gr (3 times/day), Linezolid 600 mg (2 times/day)	Ciprofloxacin 750 mg (2 times/day), Sodium Fucidate 500 mg (3 times/day)
7	Negative	Ampicillin/sulbactam 1gr (4 times/day)	Ciprofloxacin 750 mg (2 times/day), Sodium Fucidate 500 mg (3 times/day)
8	Negative	Ampicillin/sulbactam 1gr (4 times/day)	Ciprofloxacin 750 mg (2 times/day), Sodium Fucidate 500 mg (3 times/day)
9	Staphylococcus aureus (MRSA +), Streptococcus pyogenes	Teicoplanin 400 mg (1 times/day)	Ciprofloxacin 750 mg (2 times/day), Sodium Fucidate 500 mg (3 times/day)
10	Staphylococcus aureus, Diphtheroid bacilli	Rifampicin 250 mg (1 times/day), Ciprofloxacin 400 mg (2 times/day), Ertapenem 1gr (1 times/day), Teicoplanin 400 mg (1 times/day), Clindamycin 600 mg (3 times/day)	Sodium Fucidate 500 mg (3 times/day)
11	Enterobacter cloacae, Citrobacter braakii	Ertapenem 1gr (1 times/day), Teicoplanin 400 mg (1 times/day)	Cefixime 400 mg (1 time/day)
12	Klebsiella pneumoniae	Piperacillin/Tazobactam 4.5 mg (4 times/day), Ertapenem 1gr (1 times/day), Teicoplanin 400 mg (1 times/day)	Trimethoprim/sulfamethoxazole 800/160 mg (2 times/day)
13	Staphylococcus aureus (MSSA +)	Meropenem 1 gr (2 times/day)	Ciprofloxacin 750 mg (2 times/day), Sodium Fucidate 500 mg (3 times/day)
14	Negative	Teicoplanin 600 mg (1 times/day), Ciprofloxacin 400 mg (2 times/day)	Ciprofloxacin 750 mg (2 times/day), Sodium Fucidate 500 mg (3 times/day)
15	Klebsiella pneumoniae	Teicoplanin 600 mg (1 times/day), Ceftriaxone 1 gr (2 times/day)	Trimethoprim/sulfamethoxazole 800/160 mg (2 times/day), Amoxicillin 1gr (2 times/day)
16	Pseudomonas oryzihabitans, Acinetobacter baumannii	Colimycine 150 mg (2 times/day), Meropenem 1 gr (2 times/day)	Cefpodoxime 200 mg (2 times/day)
17	Staphylococcus aureus (MSSA +)	Piperacillin/Tazobactam 4.5 mg (4 times/day), Ciprofloxacin 400 mg (2 times/day)	Ciprofloxacin 750 mg (2 times/day), Sodium Fucidate 500 mg (3 times/day)
18	Negative	Teicoplanin 400 mg (1 times/day)	Ciprofloxacin 750 mg (2 times/day), Sodium Fucidate 500 mg (3 times/day)
19	Negative	Teicoplanin 400 mg (1 times/day)	Ciprofloxacin 750 mg (2 times/day), Sodium Fucidate 500 mg (3 times/day)
20	Negative	Ampicillin/sulbactam 1gr (4 times/day)	Ciprofloxacin 750 mg (2 times/day), Sodium Fucidate 500 mg (3 times/day)

At the end of the follow-up, the mean values of the patients’ SF-36 scores were as follows: PF 49.6 ± 15.7, PR 37.1 ± 30.1, BP 53.4 ± 10.1, GH 46.1 ± 5.3, VT 50.2 ± 6.1, SF 56.9 ± 14.4, ER 46.7 ± 25.9, and MH was 50.1 ± 15.1 (Table 5).

**Table 5:** Functional outcomes and quality of life of patients compared to the normal population. SF-36: Short Form-36 questionnaire. Data for the "SF‑36 Score of Normal Population" are derived from Demiral Y et al. [18].

Variables	SF‑36 score of patients (mean ± SD)	SF‑36 score of normal population (mean ± SD) [18]
Physical function	49.6 ± 15.7	83.8 ± 20.0
Physical role	37.1 ± 30.1	86.3 ± 24.9
Bodily pain	53.4 ± 10.1	82.9 ± 18.9
General health	46.1 ± 5.3	71.6 ± 16.1
Vitality	50.2 ± 6.1	64.5 ± 12.9
Social functioning	56.9 ± 14.4	91.0 ± 12.9
Emotional role	46.7 ± 25.9	90.1 ± 19.4
Mental health	50.0 ± 15.1	71.0 ± 11.0

## Discussion

The treatment of post-traumatic CO is challenging due to various factors, including difficulty in diagnosing the condition, identifying the microbiological agent, the presence of necrotic bone or implants in most patients, advanced bone pathologies, skin scars, and soft tissue defects [2-4,7,14,15]. After successful treatment, the recurrence rate of CO within one year is 30% [3,5,13,19-22]. Reported risk factors for recurrence include older age, the need for intraoperative blood transfusions, persistent symptoms for more than three months before treatment, and the presence of pseudomonal infections [5,13]. The mean follow-up period in our study was 4.5 years, and the average number of surgical procedures performed on patients was 3.1. Although we successfully treated our patients, their quality of life following CO treatment was lower compared to a normal random sample population.

Post-traumatic CO often develops from fractures, open wounds, contiguous soft-tissue infections, and orthopedic implants [1,2,5,6,12,19]. The tibia is the most common site for CO due to its higher risk of injuries, inadequate soft tissue coverage, and poor blood supply [3,12]. In our study, the tibia was the most common bone affected by CO. Regarding etiology, CO developed after an open fracture in 13 patients.

Although osteomyelitis is often polymicrobial, *Staphylococcus aureus* is the most frequently identified causative organism in CO [2,5,6,12,21]. In many cases, the infectious agent cannot be detected [2,5,21]. In our study, culture growth was detected in 13 patients, with polymicrobial growth observed in seven of these cases. Staphylococcus was the most frequently isolated agent. The tissue cultures collected were deep tissue cultures. Cultures obtained from sinus tracts may not be representative of deep pathogens, as they are often colonized by skin flora [2,7]. Therefore, diagnosis in culture-negative CO patients is usually based on clinical findings, imaging modalities, operative findings, and histology [1,2,4,21,23].

It has been reported that osteomyelitis is rarely controlled without a combination of devitalized bone and tissue removal, orthopedic device removal, and long-term (4-6 weeks) high-dose parenteral antibiotic therapy [3,6-10,16,22]. Given that revascularization of infected bone takes 3-4 weeks, CO is often treated with parenteral, high-dose, and long-term antibiotic therapy [6,7,19,22]. However, no study definitively shows the superiority of this period over others [19,22], and the duration of treatment remains empirical [19,20,22]. We administered intravenous antibiotics to our patients for an average of 6 weeks during their hospital stay and oral antibiotics for an average of 8 weeks after discharge.

Debridement involves the excision of all sequestered areas along with necrotic tissue, dead bones, sinuses, and infected granulation tissue until bleeding can be seen on the bone [1,2,4,10,14]. The final limit of debridement is determined intraoperatively by the paprika sign (point-shaped bleeding), characterized as punctate hemorrhages noted on well-vascularized living bone [4,10,14,19]. However, the macroscopic view does not always enable the identification of truly healthy tissue [10,23]. Preoperative planning based on radiographic bone scanning can be performed to identify dead spaces and infected structures [4]. Therefore, to determine the true debridement border, contrast-enhanced CT and MRI were performed on all our patients before surgery. Moreover, three patients for whom a decision could not be made regarding infection eradication after debridements were evaluated with LLTI.

Dead space from debridement can cause limb instability and susceptibility to infection [13,14,19]. Therefore, these areas need appropriate management to prevent recurrence and maintain bone integrity [12,13,19]. Dead spaces can be supported by a variety of vascularized soft tissues, whether local or distant, pedicled or free, muscular or fasciocutaneous flaps [2,8,19,24,25]. However, the superiority of flap types over each other remains controversial [2,8,19,24,25]. In our study, we applied a soleus muscle flap to two patients with tibial osteomyelitis, a local flap to two patients, and a free fascia transfer to one patient, achieving successful outcomes.

There is no clear evidence that biological and nonbiological approaches to dead space management in CO are superior to each other [21]. Antibiotic-impregnated bone cement spacers (AIBCS) are effectively used in the treatment of CO [2,4,16,26]. AIBCS is useful for filling the dead space formed after debridement and in local bone and soft tissue infections, providing antibiotic concentrations that are much higher than the minimum inhibitory concentration for most pathogens [14,26,27]. Aminoglycosides and vancomycin are the most widely used local antibiotics due to their broad spectrum, good elution properties from polymethylmethacrylate (PMMA) bone cement, and thermo-stability [4,14,16,19,26,27]. AIBCSs release very high concentrations of antibiotics while filling the dead space, preventing hematoma formation and suppuration [13,16,26]. We used 4 grams of vancomycin for every 40 grams of PMMA bone cement after debridement in all patients. We used AIBCS not as beads, but as a void filler to fill the entire defective area. PMMA removal was not required in seven patients, as we did not encounter any problems in these cases. Bor N et al. advocated for the long-term use of AIBCSs in a study of 16 patients with an average of six years of follow-up, reporting that it was an advantage not to require a second surgery, especially in elderly patients with comorbidities [26]. Fernando N et al. reported no wound complications or recurrent infections in 37 patients who did not undergo removal of AIBCSs at long-term follow-up [28]. Similarly, Qiu XS et al. reported that AIBCSs were not removed following treatment for CO in eight patients, and no signs of osteomyelitis recurrence were observed after a mean follow-up of two years [29].

Re-debridement greatly reduces the cortical volume, increasing the risk of iatrogenic fractures [4,11,15]. Therefore, after debridement, the anatomical stability of the fracture site should be restored as much as possible [4,11,15]. External fixations have become a standard method of skeletal stabilization after surgery for CO, due to concerns about the occurrence of infection in any internal fixation [10,11,15]. For this reason, we preferred to use a prophylactic monolateral or Ilizarov external fixator for a certain period to prevent pathological fracture and protect the bone. After healing, it is important that the bone is stable enough to allow weight-bearing [11,15]. Therefore, we applied various surgical procedures such as monofocal-bifocal bone transport, the Masquelet technique, free vascularized and nonvascularized fibular grafting, autograft-allograft application, plate osteosynthesis, and intramedullary nailing for stabilization, according to the final clinical status of the extremities of the patients. All these surgical methods were performed not as a standard procedure, but considering the response to the treatments applied based on the pathologies of the patients from the start of the treatment.

This study had some limitations. First, the number of patients was relatively small. However, it should be taken into consideration that CO is not very common and the treatment process is also complex and long-standing. The treatment process of each patient in our study is a case report in itself. Second, this study did not have a comparison group. There is no single method that can be applied to every patient in the treatment of CO. The aim of our study was to apply treatment combinations specific to the differences in each patient's pathology.

## Conclusions

We described the long-term clinical and functional outcomes of patients with post-traumatic C-M type III and IV CO, who were managed considering individual differences, leading to successful outcomes. Many treatment procedures have been described for CO. Due to the varied histories of CO and individual differences, it is quite challenging to plan a standard treatment procedure for CO in clinical practice. Successful treatment can be achieved using a long-term multidisciplinary approach, with individualized and well-planned treatment methods. The development of CO treatment methods can be advanced with this and similar studies, fostering new ideas and more treatment options.

## References

[1] Hogan A, Heppert VG, Suda AJ (2013). Osteomyelitis. Arch Orthop Trauma Surg.

[2] Chan JK, Ferguson JY, Scarborough M, McNally MA, Ramsden AJ (2019). Management of post-traumatic osteomyelitis in the lower limb: current state of the art. Indian J Plast Surg.

[3] Arshad Z, Lau EJ, Aslam A, Thahir A, Krkovic M (2021). Management of chronic osteomyelitis of the femur and tibia: a scoping review. EFORT Open Rev.

[4] Yang J, Yao JL, Wu ZQ (2021). Current opinions on the mechanism, classification, imaging diagnosis and treatment of post-traumatic osteomyelitis. Chin J Traumatol.

[5] Jorge LS, Chueire AG, Fucuta PS, Machado MN, Oliveira MG, Nakazone MA, Salles MJ (2017). Predisposing factors for recurrence of chronic posttraumatic osteomyelitis: a retrospective observational cohort study from a tertiary referral center in Brazil. Patient Saf Surg.

[6] Mormeneo Bayo S, Ferrer Cerón I, Martín Juste P, Lallana Dupla J, Millán Lou MI, García-Lechuz Moya JM (2020). A review of difficult-to-treat post-traumatic osteomyelitis: Role of Clostridium celerecrescens. Rev Esp Cir Ortop Traumatol (Engl Ed).

[7] Heitzmann LG, Battisti R, Rodrigues AF, Lestingi JV, Cavazzana C, Queiroz RD (2019). Postoperative chronic osteomyelitis in the long bones - current knowledge and management of the problem. Rev Bras Ortop (Sao Paulo).

[8] Thai DQ, Jung YK, Hahn HM, Lee IJ (2021). Factors affecting the outcome of lower extremity osteomyelitis treated with microvascular free flaps: an analysis of 65 patients. J Orthop Surg Res.

[9] Koyuncu Ş, Ozan F (2012). Morganella morganii osteomyelitis complicated by secondary septic knee arthritis: a case report. Acta Orthop Traumatol Turc.

[10] Fodor L, Ullmann Y, Soudry M, Calif E, Lerner A (2006). Prophylactic external fixation and extensive bone debridement for chronic osteomyelitis. Acta Orthop Belg.

[11] Kliushin NM, Burnashov SI, Mekki WA, Leonchuk DS, Sudnitsyn AS (2022). Treatment of postoperative tibial chronic osteomyelitis using bone transport techniques; an observational study. J Clin Orthop Trauma.

[12] Zhou CH, Ren Y, Ali A, Meng XQ, Zhang HA, Fang J, Qin CH (2020). Single-stage treatment of chronic localized tibial osteomyelitis with local debridement and antibiotic-loaded calcium sulfate implantation: a retrospective study of 42 patients. J Orthop Surg Res.

[13] Rodham P, Panteli M, Qin C, Harwood P, Giannoudis PV (2023). Long-term outcomes of lower limb post-traumatic osteomyelitis. Eur J Trauma Emerg Surg.

[14] Wu H, Shen J, Yu X, Fu J, Yu S, Sun D, Xie Z (2017). Two stage management of Cierny-Mader type IV chronic osteomyelitis of the long bones. Injury.

[15] Zhou CH, Ren Y, Song HJ (2021). One-stage debridement and bone transport versus first-stage debridement and second-stage bone transport for the management of lower limb post-traumatic osteomyelitis. J Orthop Translat.

[16] Masquelet AC, Begue T (2010). The concept of induced membrane for reconstruction of long bone defects. Orthop Clin North Am.

[17] Cierny G 3rd, Mader JT, Penninck JJ (2003). A clinical staging system for adult osteomyelitis. Clin Orthop Relat Res.

[18] Demiral Y, Ergor G, Unal B, Semin S, Akvardar Y, Kivircik B, Alptekin K (2006). Normative data and discriminative properties of short form 36 (SF-36) in Turkish urban population. BMC Public Health.

[19] Haidar R, Der Boghossian A, Atiyeh B (2010). Duration of post-surgical antibiotics in chronic osteomyelitis: empiric or evidence-based?. Int J Infect Dis.

[20] Gomes D, Pereira M, Bettencourt AF (2013). Osteomyelitis: an overview of antimicrobial therapy. Braz J Pharm Sci.

[21] Pincher B, Fenton C, Jeyapalan R, Barlow G, Sharma HK (2019). A systematic review of the single-stage treatment of chronic osteomyelitis. J Orthop Surg Res.

[22] Spellberg B, Lipsky BA (2012). Systemic antibiotic therapy for chronic osteomyelitis in adults. Clin Infect Dis.

[23] Govaert GA, IJpma FF, McNally M, McNally E, Reininga IH, Glaudemans AW (2017). Accuracy of diagnostic imaging modalities for peripheral post-traumatic osteomyelitis - a systematic review of the recent literature. Eur J Nucl Med Mol Imaging.

[24] Salgado CJ, Mardini S, Jamali AA, Ortiz J, Gonzales R, Chen HC (2006). Muscle versus nonmuscle flaps in the reconstruction of chronic osteomyelitis defects. Plast Reconstr Surg.

[25] Paro J, Chiou G, Sen SK (2016). Comparing muscle and fasciocutaneous free flaps in lower extremity reconstruction--does it matter?. Ann Plast Surg.

[26] Bor N, Dujovny E, Rinat B, Rozen N, Rubin G (2022). Treatment of chronic osteomyelitis with antibiotic-impregnated polymethyl methacrylate (PMMA) - the Cierny approach: is the second stage necessary?. BMC Musculoskelet Disord.

[27] Springer BD, Lee GC, Osmon D, Haidukewych GJ, Hanssen AD, Jacofsky DJ (2004). Systemic safety of high-dose antibiotic-loaded cement spacers after resection of an infected total knee arthroplasty. Clin Orthop Relat Res.

[28] Fernando N, Werner S, Elhaddad M, Davies J, Firoozabadi R (2020). Do antibiotic beads need to be removed?. Arch Bone Jt Surg.

[29] Qiu XS, Zheng X, Shi HF (2015). Antibiotic-impregnated cement spacer as definitive management for osteomyelitis. BMC Musculoskelet Disord.

